# Inhibition of astrocyte signaling leads to sex-specific changes in microglia phenotypes in a diet-based model of small cerebral vessel disease

**DOI:** 10.21203/rs.3.rs-6198453/v1

**Published:** 2025-03-17

**Authors:** Jenna L. Gollihue, Khine Zin Aung, Colin B. Rogers, Pradoldej Sompol, Yuriko Katsumata, Erica M. Weekman, Donna M. Wilcock, Josh M. Morganti, Christopher M. Norris

**Affiliations:** University of Kentucky; University of Kentucky; University of Kentucky; University of Kentucky; University of Kentucky; Indidana University School of Medicine; Indidana University School of Medicine; University of Kentucky; University of Kentucky

**Keywords:** Astrocyte reactivity, neuroinflammation, calcium, vascular, microglia

## Abstract

Hyperhomocysteinemia (HHcy)-inducing diets recapitulate small cerebral vessel disease phenotypes in mice including cerebrovascular pathology/dysfunction, neuroinflammation, synaptic deficits, and cognitive decline. We recently showed that astrocyte signaling through calcineurin(CN)/nuclear factor of activated T cells (NFATs) plays a causative role in these phenotypes. Here, we assessed the impact of astrocytic signaling on microglia, which set inflammatory tone in brain. Seven-to-eight-week-old male and female C57BL/6J mice received intrahippocampal injections of AAV2/5-Gfa2-EGFP (control) or adeno-associated virus (AAV) expressing the NFAT inhibitor VIVIT (*i.e.,* AAV2/5-Gfa2-VIVIT-EGFP). Mice were then fed with control chow (CT) or B-vitamin-deficient chow for 12 weeks to induce HHcy. Immunohistochemistry was used to assess the expression of the pan-microglial marker Iba1 and the homeostatic microglial marker P2ry12. Iba1 showed little sensitivity to diet, AAV treatment, or sex. Conversely, P2ry12 expression was reduced with HHcy diet in males, but not females. Treatment of males with AAV-Gfa2-VIVIT prevented the loss of P2ry12. We next conducted single-cell RNA sequencing (scRNAseq) to determine if microglial genes and/or microglial clustering patterns were sensitive to astrocyte signaling in a sex-dependent manner. In males, disease-associated microglial genes and subclusters were overrepresented in HHcy-treated mice, while VIVIT promoted the appearance of homeostatic microglial genes and clusters. In contrast, microglial genes in females were less sensitive to diet and AAV treatments, though disease-like patterns in gene expression were also observed in the HHcy condition. However, very few of the HHcy-sensitive microglial genes in females were affected by VIVIT. The results suggest a sexually dimorphic influence of astrocyte signaling on microglial phenotypes in the context of HHcy and small cerebral vessel disease.

## INTRODUCTION

Cerebral small vessel disease is a major vascular contribution to cognitive impairment and dementia (VCID) and commonly appears with Alzheimer’s disease (AD) and related dementias (ADRD)[1–3]. Hyperhomocysteinemia (HHcy) can drive several types of vascular pathologies and is an independent risk factor for both stroke and AD[4, 5]. Diets deficient in B6, B12, and folate, and enriched in methionine, give rise to HHcy and recapitulate numerous phenotypes of VCID including microhemorrhages neuroinflammation, altered gene expression, impaired cerebral blood flow, reduced neurovascular coupling, and cognitive loss[6–10]. Moreover, HHcy potentiates AD-related pathologies and/or limits functional improvements typically found with anti-amyloid treatments[11, 12]. Work from our groups have suggested that many of the adverse effects of HHcy on brain structure and function may arise from altered astrocyte signaling [7, 10].

Reactive astrocytes are a heterogeneous population of macroglia that can appear prominently with aging, injury, and chronic neurodegenerative conditions including ADRDs like VCID[13–16]. Because astrocytes interact with most cell types in the brain either directly, or indirectly, reactive astrocyte signaling has the potential to have a far-reaching impact on brain structure and function. To determine specific effects of astrocyte signaling in intact rodent models of AD and ADRDs, several groups have turned to cell-specific targeting approaches to inhibit transcription factor pathways including NFATs[17–19], STATs[20, 21], and NFκB[22] that drive or maintain astrocyte reactivity. In addition to assessing the impact of reactive astrocytes on disease biomarkers (*e.g.,* Aβ and Aβ deposits), cell-specific targeting approaches used by these groups have revealed largely detrimental effects of reactive astrocytes on neurons and synapses in AD models. Inhibition of reactive astrocyte signaling (*i.e.,* NFAT activity) was also recently found to preserve synaptic function and plasticity in an HHcy model of VCID, in conjunction with improvements of neurovascular coupling, capillary blood flow, and cerebral perfusion[10].

While these reports show that reactive astrocytes can significantly disrupt the structure and function of the neurovascular unit in AD and ADRD models, less is known about the impact of reactive astrocytes on other major cell types in the brain. Previously, we found that inhibition of astrocyte NFATs in rodent models either reduced[17] or had little effect[18] on common microglial protein markers, depending on the pathological context (*i.e.,* chronic amyloid pathology vs. acute injury), suggesting that astrocyte-microglial interactions are nuanced To explore these nuances further, we used immunohistochemical and single-cell RNA sequencing (scRNAseq) approaches to assess the impact of astrocytic NFAT signaling on microglia phenotypes after diet-mediated induction of HHcy. The results suggest that reactive astrocytes affect microglia differently in males and females during progression of HHcy.

## METHODS

### Mice/surgery

Mice used here were part of a previously described cohort that was investigated to assess effects of HHcy diet and astrocytic NFAT activity on behavioral status[10]. All animals were treated in accordance with the National Institutes of Health Guide for the Care and Use of Laboratory animals and the University of Kentucky IACUC animal welfare guidelines. C57BL/6J mice (Jackson labs) were group housed in 12hr light/dark cycle with access to food and water ad libitum. Per our previous work, AAV 2/5 vectors encoding the human GFAP (Gfa2) promotor was used to specifically drive transgene expression in astrocytes[10, 17–19]. At 8 weeks of age, male and female mice underwent injections of either control AAV2/5-Gfa2-EGFP or AAV2/5-Gfa2-VIVIT bilaterally into the hippocampus. Briefly, animals were anesthetized using 2.5% isoflurane (Somnosuite, Kent scientific) and head-fixed using a stereotaxic frame. After incision over the skull, coordinates for the hippocampus were marked followed by a small craniotomy. A microinjector (Stoelting) with a 10μL Hamilton syringe was used to inject 4 μL of AAV into the brain at a rate of 0.2 μl/min. The needle was then slowly removed and the incision site was closed using absorbable sutures. Following surgery, animals were returned to their home cage for recovery. Three weeks after injection, and allowing for full expression of the AAV, mice were put on either a control diet or HHcy diet for 12 weeks. The HHcy diet (Harlan Teklad TD97345; Harlan Teklad, Madison, WI, USA) is enriched in methionine, but contains low levels of folate, vitamin B6, and vitamin B12. Control diet (Harlan Teklad 5001C; Harlan Teklad) had normal levels of methionine, folate, B6, and B12. Mice used in this cohort were assessed in the radial arm water maze and in an open field maze and are described in our recent work[10], in which HHcy led to cognitive impairments that were ameliorated by AAV-Gfa2-VIVIT. At approximately 1 week after behavioral assessment, a small cohort of mice were further processed to evaluate microglia using immunohistochemical labeling and scRNAseq.

Immunohistochemistry: Thirty mice were used for immunohistochemical labeling: Eight CT diet EGFP (four males and four females), eight CT diet VIVIT (four males and four females), eight HHcy diet EGFP (four males and four females), six HHcy diet VIVIT (four males and two females). One week after behavioral assessment (at 24 weeks old), mice were transcardially perfused with PBS. Brains were removed and post-fixed in 4% paraformaldehyde for 24 hours, then transferred to 30% sucrose in PBS until tissues sank. Brain sections were then cut on a freezing stage cryotome at 40um thickness. Free floating sections were selected and washed 3X with PBS. This was followed by blocking peroxidase activity (30 min in 0.3% H_2_O_2_ in methanol), washing 3X in PBS, blocking for 1 hour (10% goat serum, 0.2% triton × 100 in PBS), and overnight incubation with the primary antibody (microglia- P2RY12 1:2000 Anaspec #AS-55043A, IBA1 1:10,000 HistoSure #HS-234308). On day 2, the sections were washed 3X in PBS (P2RY12) or TBS (IBA1), incubated in secondary antibody for 1 hour at room temp (1:200 goat anti-rabbit biotinylated or 1:500 goat anti-Guinea pig biotinylated), washed 3X in PBS (P2RY12) or TBS (IBA1), incubated in ABC solution for 1 hour according to manufacturer instructions (ABC Elite, Vector #PK-6100), washed 3X in PBS (P2RY12) or TBS (IBA1), followed by incubation in DAB (Vector cat# SK-4100). Following exposure to DAB, the sections were quickly quenched in H_2_O, and washed 5X in PBS. The sections were then mounted onto slides and dried overnight before dehydrating in sequential ethanol and safeclear, and coverslipping with DPX mounting media. Slides were imaged on the Nikon BioPipeline Slide Scanner at 10X. The images were analyzed using HALO software (Indica Labs). A region of interest was drawn around the hippocampus and measurements were taken for the percentage of ROI positively stained. Two sections per mouse were analyzed. For statistical analyses, the sample size was equal to the number of sections per group.

### Cell type isolation for scRNAseq

Pooled brain tissue (n=2 per experimental group) was used to generate ‘glia-enriched’ single cell suspensions as previously described[23]. Briefly, mice were anesthetized via 5.0% isoflurane before exsanguination and transcardial perfusion with ice-cold Dulbecco’s phosphate buffered saline (DPBS; Gibco # 14040133). Following perfusion, brains were quickly removed, hippocampi dissected from each hemisphere and pooled, then quickly minced using forceps on top of an ice-chilled petri dish. Minced tissue was immediately transferred into gentleMACS C-tube (Miltenyi #130–093–237) containing Adult Brain Dissociation Kit (ADBK) enzymatic digest reagents (Miltenyi #130–107–677) prepared according to manufacturer’s protocol. Tissues were dissociated using the “37C_ABDK” protocol on the gentleMACS Octo Dissociator instrument (Miltenyi #130–095–937) with heaters attached. After tissue digestion, cell suspensions were filtered through 70 μm mesh cell filters to remove debris following the manufacturer’s suggested ABDK protocol. The resultant suspension was sequentially filtered (x2) using fresh 30 μm mesh filters. Cell viability was checked using AO/PI viability kit (Logos Biosystems # LGBD10012). All cell suspensions were determined to have > 90% viable cells. Following viability and counting, cells were diluted to achieve a concentration of ~ 1700 cells/μL in a 10μL total reaction volume. The diluted cell suspensions were loaded onto the 10X Chromium Connect automated cell portioning system. Sample libraries were constructed using NextGEM automated 3’ reagents (10X Genomics, v3.1) following manufacturer’s suggested protocol (#CG000286 Rev B). Final library quantification and quality check was performed using BioAnalyzer (Agilent), and sequencing performed on a NovaSeq 6000 S4 flow cell, 150 bp Paired-End sequencing (Novogene).

### Computational scRNAseq data post sequencing processing

The Cell Ranger 8.0.1 workflow was used to align paired-end reads formatted in two FASTQ files to the Genome Reference Consortium Mouse Build 39 (GRm39)[24]. Then, raw unique molecular identifier (UMI) count data for each sample were generated using the “cellranger count” function containing the number of reads for genes in each cell per sample. Based on immunohistochemistry results, which indicated sex-specific differences in the response of microglial protein markers to both HHcy diet and AAV-Gfa2-VIVIT treatment, subsequent scRNAseq analyses were performed for male and female mice separately. Four UMI count matrixes for females and four UMI count matrixes for males were loaded as Seurat objects using the “CreateSeuratObject” function with a minimum threshold of 3 cells and 200 detected genes implemented by the “Seurat” R (version 5.1.0) R package[25–27]. The percentages of mitochondrial contents were calculated in each cell using the “PercentageFeatureSet” function with pattern matching with mitochondrial genes (*i.e.,* pattern = “^mt-”). We filtered out cells with the lower 0.5% and upper 99.5% quantiles of gene expression and cells with more than 20% mitochondrial genes to reduce the potential of including low quality cells and droplets. Mitochondrial and ribosomal genes were removed because high expression of those genes can disproportionately represent stressed or apoptotic cells, and mask cell-type-specific signals essential for meaningful biological interpretation in scRNAseq analyses[28]. The filtered UMI count matrix data were normalized using the “NormalizeData” function with the default method (*i.e.,* normalization.method = LogNormalize) and 2000 highly variable genes were identified to capture significant biological differences between cells using the “FindVariableFeatures” function with the “vst” method.

In order to integrate the Seurat objects within each sex, we first identified highly variable genes across individual cells using the “SelectIntegrationFeatures” function, second applied the “FindIntegrationAnchors” function to identify anchor cells across the Seurat objects, and then used the “IntegrateData” function which aligned and integrated the four Seurat objects. The normalized UMI counts for each gene in the integrated Seurat object were scaled using the “ScaleData” function to center each gene expression at a mean of 0 and its variance to 1.

### Cell type identification

We first performed Uniform Manifold Approximation and Projection (UMAP) using the “RunUMAP” function with n.neighbors = 30, min.dist = 0.5, and method = “euclidean based on the first 20 principal components (PCs) generated with the “RunPCA” function. Next, we used “FindNeighbors” with the default setting to calculate the neighborhood overlap (Jaccard index) between cells based on the first 20 PCs to identify relationships with similar cells. Then, we defined clusters using the “FindClusters” function with a resolution of 0.4. To determine cell types for each cluster, Spearman correlations were calculated to assess cluster similarities and were visualized as a clustered heatmap using the “pheatmap” (version 1.0.12) R package (Supplementary Fig. 1, with an example of cell type identification for male mice). We also visualized gene expressions of selected maker genes which were identified from the previous study[23] for each cell type in two dimensional UMAP plot using the “FeaturePlot” function. In addition, we used the FindConservedMarkers function to find the most biologically meaningful marker genes [false discovery rate (FDR) adjusted p-value < 0.05 and a log2 fold change > 0.5 across different conditions] for other cell types not described in this study[23].

### Differential expressed gene analysis

We performed differential expressed gene analyses across different experimental groups using a zero-inflated negative binomial (ZINB) regression model implemented by the “zinbwave” (version 1.20.0)[29] and “edgeR” (version 3.40.2)[30] Bioconductor R packages. After we filtered out low-expressed genes (those who expressed less than 25% of the total number of cells), we fitted a ZINB model using the “zinbFit” function to account for zero inflation and overdispersion. Then, we conducted the differential gene expression analyses using the edgeR R package. The normalization factors were calculated using the “calcNormFactors” to normalize the library sizes across samples. The observational weights computed from the “zinbwave” function in the “zinbwave” R package were integrated into the analyses. The negative binomial dispersions were estimated with the “estimateDisp” function and the ZINB models were fitted with the design matrix representing the four experimental groups using the “glmFit” function. The F-tests with adjusted degree of freedom for ZINB were performed to identify differentially expressed genes based on a threshold of FDR-adjusted P value < 0.05.

### Enrichment Analysis

Significant genes with false discovery rate adjusted p-value < 0.05 and absolute value of log_2_ fold change ≥ 0.2 were included in the Kyoto Encyclopedia of Genes and Genomes (KEGG) enrichment analyses which were performed using the “enrichKEGG” function in the “clusterProfiler” (version 4.6.2) Bioconductor R package [31].

### Sub-clustering

After subtracting the microglia population from the main cell clusters, we performed normalization, integration, and sub-clustering (described above). Then, we used “FindAllMarkers” and filtered genes expressed in at least 25% of cells and a minimum log_2_ fold change of 0.2. We then used known disease-related microglia markers (Apoe, H2-D1, H2-K1, B2m, and Csf1) and homeostatic microglia markers (Gpr34, P2ry12, Fcrls, Tmem119, and Siglech) to assign the clusters into disease-related microglia cluster and homeostatic microglia cluster for both male and female mice.

### Statistical Analyses

A three-way analysis of variance (ANOVA) was used to assess effects of diet, AAV treatment, and sex on Iba1 and P2ry12 immunolabeling levels in hippocampus. Significant interaction effects were followed by tests of simple main effects within males and females, followed by Dunnett’s Multiple Comparison’s test to evaluate changes in treatment groups relative to the CT diet EGFP condition. Significance was set at p < 0.05.

## RESULTS

### HHcy diet and VIVIT treated affect P2ry12 expression differently in males and females

**Figure 1:**
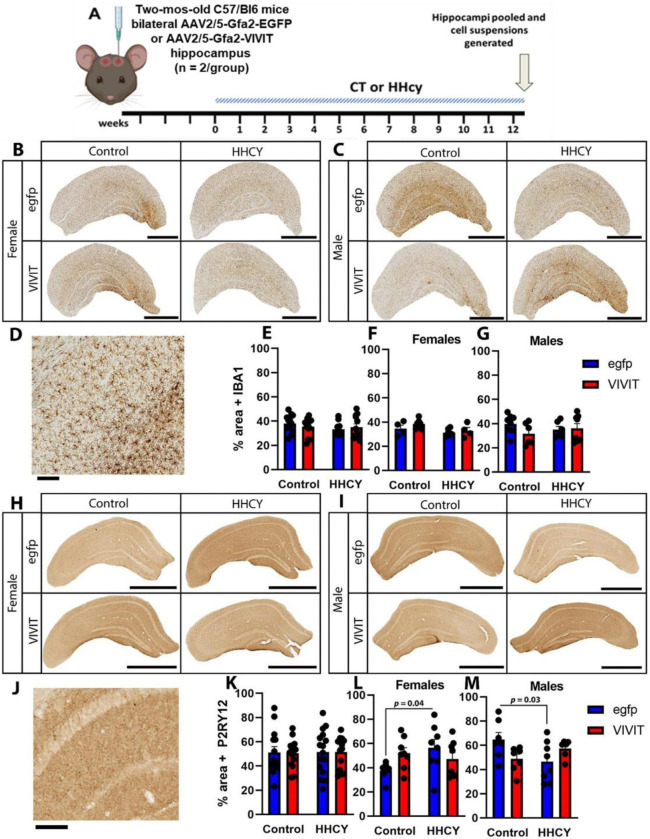
HHcy diet and VIVIT treatment alter Iba1 and P2ry12 immunolabeling in sex dependent manner. **A,** time-line of diet and AAV treatments. Male and female Mice received bilateral hippocampal injections of AAV vectors at approximately two months of age. One month following AAV injection, mice were started on a control (CT) diet or a HHcy inducing diet. At 12–15 weeks of diet treatment, mice were harvested and brains were sections for immunolabeling or scRNA seq analyses. **B-C**, low magnification photomicrographs of Iba1 immunolabeling in hippocampus across the four diet/AAV conditions in female (**B**) and male (**C**) mice. calibration bar is 1 mm. **D**, Iba1 immunolabeling photo micrograph at higher magnification. calibration bar is 100 μm. **E-G**, % area occupied by lba1 immunolabel across all mice (**E**), only females (**F**), or only males (**G**). H-I, Iow magnification photomicrographs of P2ry12 immunolabeling in hippocampus across the four diet/AAV conditions in female (**H**) and male (**I**) mice. calibration bar is 1 mm. **J**, P2ry12 immunolabeling photo micrograph at higher magnification. calibration bar is 100 μm. **K-M**, % area occupied by P2ry12 immunolabel across all mice (**K**), only females (**L**), or only males (M). HHcy had opposing effects on P2ry12 labeling in males and females (determined by three way ANOVA and Dunnett’s Multiple Comparison’s test. Each plot symbol represents an individual brain section.

At eight weeks of age, C57BL/6J mice received intrahippocampal injections of AAV-Gfa2 vectors (Fig. 1A) to drive astrocyte specific expression of EGFP (control) or VIVIT (NFAT inhibitor), per our previous work on mice[10, 17, 19] and rats[18]. Three weeks later, mice were fed control diet (CT) or HHcy-inducing diet for 12 weeks. The four treatment groups were CT-diet with AAV-Gfa2-EGFP (CT EGFP), CT-diet with AAV-Gfa2-VIVIT (CT VIVIT), HHcy-diet with AAV-Gfa2-EGFP (HHcy EGFP), and HHcy-diet with AAV-Gfa2-VIVIT (HHcy VIVIT). Brain sections were labeled for the pan reactive microglial marker Iba1 (Fig. 1B,C low magnification; Fig. 1D high magnification) and the homeostatic microglial marker P2ry12 (Fig. 1H,I low magnification; Fig. 1J high magnification). Iba1 levels across all mice are shown in Fig. 1E, and levels for females and males are plotted separately in Fig. 1F and 1G. No significant effects of sex, diet, or AAV treatment were observed for Iba1 expression. P2ry12 levels across all mice are shown in Fig. 1K, and levels for females and males are plotted separately in Fig. 1L and 1M. A three way ANOVA detected a significant sex × diet × AAV interaction [*F*(1,51)=12.08,p=0.001]. Tests for simple main effects within males and females each revealed significant diet x AAV interactions [females F(1,27) = 5.255, p = 0.03; males F (1,24) = 7.05, p = 0.01]. P2ry12 levels in female CT EGFP mice (Fig. 1L) were reduced relative to their male counterparts (Fig. 1M, CT EGFP). Differences between the CT EGFP and HHcy EGFP groups were in opposite directions depending on sex. For females, HHcy resulted in a significant increase in P2Ry12 levels (p = 0.04), while in males, HHcy resulted in a significant reduction in HHcy levels (p = 0.03).

### Effects of HHcy diet and VIVIT treatment on microglial gene expression in females

**Figure 2:**
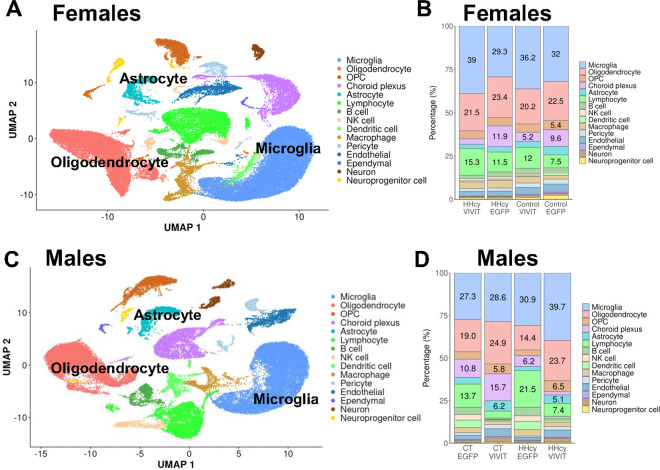
Cell clusters in males and females based on canonical gene expression markers. UMAPs show 15 unique cell clusters in female (**A**) and male (**C**) mice. The corresponding proportions of each unique cluster is shown in panel **B** (for females) and panel **D** for males.

To determine if molecular phenotypes of microglia are also affected by sex, diet, and AAV treatment conditions, we compared relative gene expression levels and microglial clustering patterns using scRNA-seq. We identified 15 unique clusters that were assigned cell-type identifiers based on established gene expression profiles (Fig. 2). Microglia, identified using a panel of markers (*e.g.,* Cx3cr1, P2ry12, Trem2, and Csf1r, Supplementary Fig. 1) was the most abundant cell type for females (Figs. 2A and B) and males (Fig. 2C and 2D) across diet and AAV treatment conditions.

**Figure 3:**
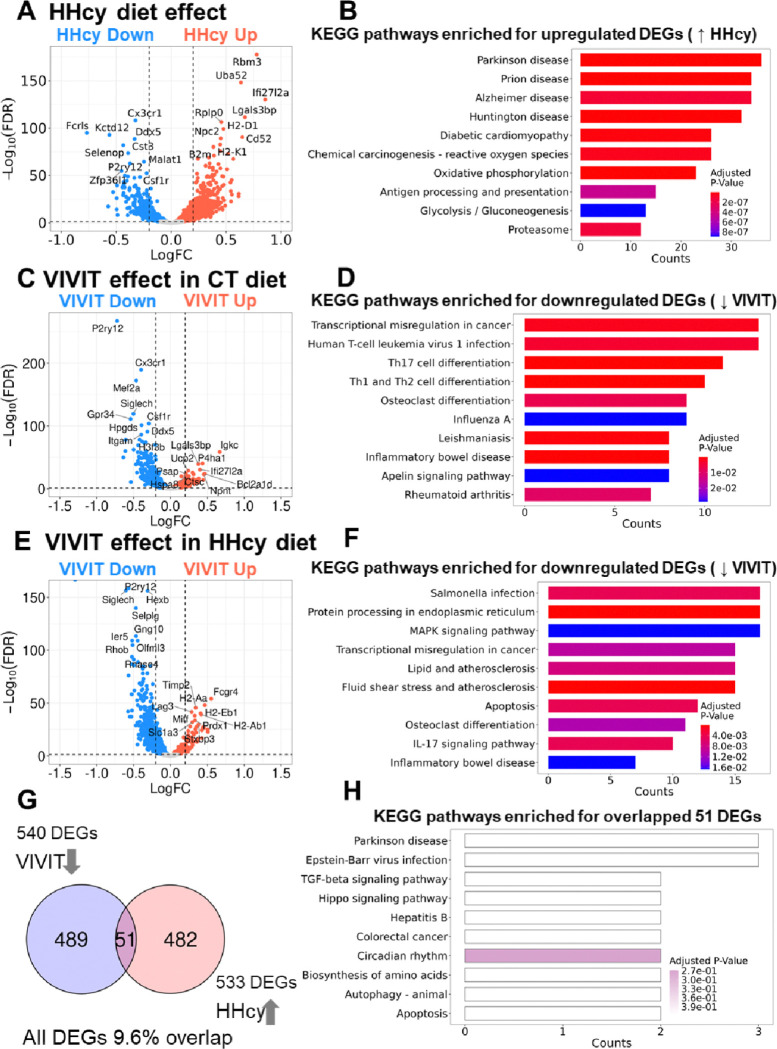
Differentially expressed microglial genes and KEGG pathway mapping in females. A, Volcano plot shows microglial DEGs that are upregulated (HHcy Up, red) and downregulated (HHcy Down, blue) in the HHcy-EGFP vs CT EGFP treatment conditions. B, KEGG pathways enriched for HHcy-sensitive (upregulated) DEGs. C, Volcano plot shows microglial DEGs that are upregulated (VIVIT Up, red) and downregulated (VIVIT Down, blue) by VIVIT in the CT-EGFP vs CT VIVIT treatment conditions. D, KEGG pathways enriched for VIVIT-sensitive (downregulated) DEGs in the CT EGFP vs CT VIVIT conditions. E, Volcano plot shows microglial DEGs that are upregulated (VIVIT Up, red) and downregulated (VIVIT Down, blue) by VIVIT in the HHcy-EGFP vs HHcy VIVIT treatment conditions. F, KEGG pathways enriched for VIVIT-sensitive (downregulated) DEGs in the HHcy EGFP vs HHcy VIVIT conditions. G, Venn diagrams showing DEGs that are sensitive to both HHcy (533 genes upregulated in HHcy EGFP vs CT EGFP comparison) and VIVIT (540 genes downregulated in HHcy VIVIT vs HHcy EGFP). Very little overlap (51 genes) was observed for HHcy and VIVIT sensitive genes.

In females, the effects of diet were first assessed in EGFP-treated mice (CT EGFP vs HHcy EGFP). The volcano plot in Fig. 3A, shows microglial DEGs that were upregulated (red plot symbols) and downregulated (blue plot symbols) by HHcy. In apparent contrast to the immunolabeling results (Fig. 1L), many of the DEGs that were upregulated by HHcy (> 480 DEGs, with a log fold change of 0.2 and FDR < 0.05) have been previously identified as disease-associated microglial (DAM) markers (*e.g.,* H2-D1, H2-K1, B2m, and others, see Supplementary Table 1 for full list of HHcy-sensitive DEGs). In contrast, many of the genes downregulated with HHcy treatment (> 200 genes, with a log fold change of 0.2 and FDR < 0.05) have been linked to homeostatic microglia (HSMG) phenotypes (*e.g.,* P2ry12, Cst3, Fcrls and others, see Supplementary Table 1, for full list). Upregulated HHcy sensitive genes were enriched in functional pathways (Fig. 3B) linked to multiple neurodegenerative diseases (*e.g.,* Parkinsons disease, Prion disease, Alzheimer disease, and Huntington disease), cardiomyopathy, alterations in oxidative phosphorylation and oxidative stress, and antigen processing/presentation.

Treatment of female mice with VIVIT tended to result in the downregulation of microglial genes, regardless of diet treatment. Volcano plots for the CT diet (CT EGFP vs CT VIVIT) and HHcy diet (HHcy EGFP vs HHcy VIVIT) groups are shown in Fig. 3C and 3E, respectively. Many of the downregulated VIVIT-sensitive genes included homeostatic microglia (HSMG) markers like P2ry12, Cx3cr1, Siglech and others (Fig. 3C and 3E, see Supplementary Tables 2 and 3 for full lists of VIVIT-sensitive DEGs). KEGG analyses of VIVIT sensitive genes (*i.e.,* downregulated) in the CT diet conditions (Fig. 3D) found enrichment in pathways for transcriptional misregulation in cancer, viral infections (*e.g.,* Human T-cell leukemia virus 1 and influenza A), differentiation (*e.g.,* Th1, Th2, and Th17, and osteoclasts), leishmaniasis, and inflammatory bowel disease. In the HHcy diet condition, KEGG pathways enriched for VIVIT sensitive (downregulated) genes (Fig. 3F) included Salmonella infection, protein processing in endoplasmic reticulum, MAP kinase signaling, transcriptional misregulation in cancer, atherosclerosis (related to lips and shear stress), and apoptosis. Finally, there was very little overlap (Fig. 3G) between the genes that were upregulated by HHcy (HHcy EGFP vs CT EGFP) and genes that were downregulated by VIVIT (HHcy VIVIT vs HHcy EGFP) (Fig. 3G), suggesting that the two treatments affected entirely different transcriptional programs. The genes that did overlap (51 in total) were most strongly related to circadian rhythm (Fig. 3H).

### Effects of HHcy diet and VIVIT treatment on microglial gene expression in males

**Figure 4:**
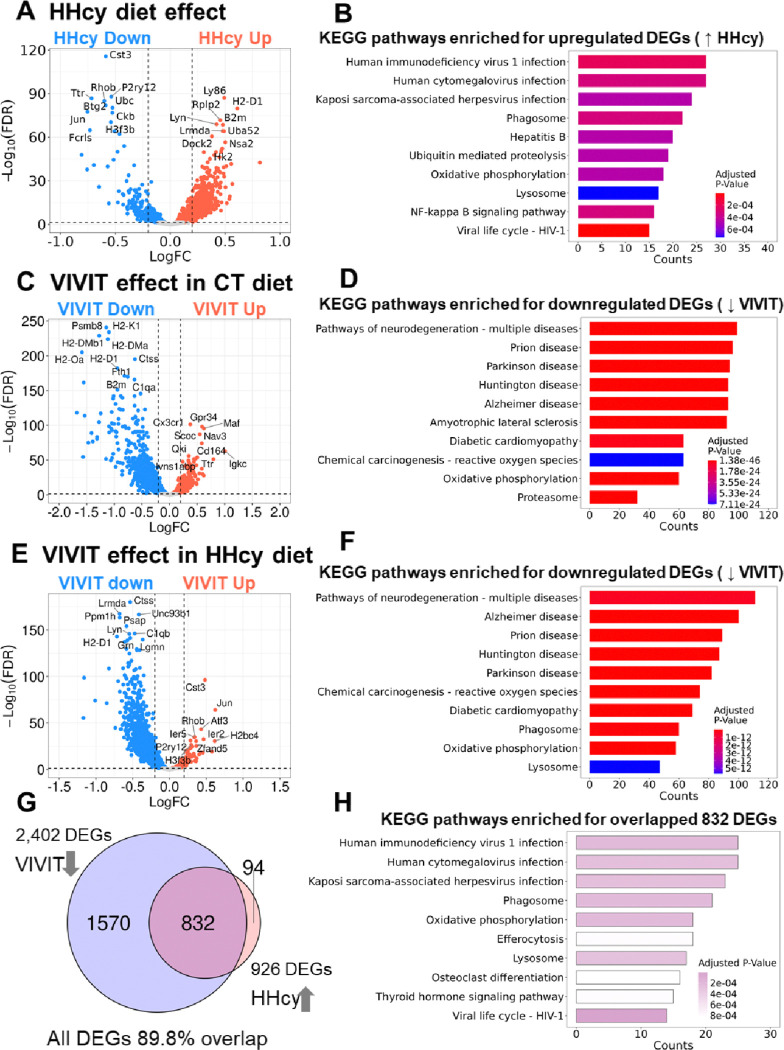
Differentially expressed microglial genes and KEGG pathway mapping in males. **A,** Volcano plot shows microglial DEGs that are upregulated (HHcy Up, red) and downregulated (HHcy Down, blue) in the HHcy-EGFP vs CT EGFP treatment conditions. **B**, KEGG pathways enriched for HHcy-sensitive (upregulated) DEGs. **C**, Volcano plot shows microglial DEGs that are upregulated (VIVIT Up, red) and downregulated (VIVIT Down, blue) by VIVIT in the CT-EGFP vs CT VIVIT treatment conditions. **D**, KEGG pathways enriched for VIVIT-sensitive (downregulated) DEGs in the CT EGFP vs CT VIVIT conditions. **E**, Volcano plot shows microglial DEGs that are upregulated (VIVIT Up, red) and downregulated (VIVIT Down, blue) by VIVIT in the HHcy-EGFP vs HHcy VIVIT treatment conditions. **F**, KEGG pathways enriched for VIVIT-sensitive (downregulated) DEGs in the HHcy EGFP vs HHcy VIVIT conditions. **G**, Venn diagrams showing DEGs that are sensitive to both HHcy (926 genes upregulated in HHcy EGFP vs CT EGFP comparison) and VIVIT (2402 genes downregulated in HHcy VIVIT vs HHcy EGFP). More than 90% of the HHcy sensitive genes (832 genes) were also sensitive to VIVIT treatment.

Gene expression patterns in males were both similar and dissimilar to what was observed in females (Fig. 4). Similarities included the directionality of gene changes in CT EGFP vs HHcy EGFP conditions (Fig. 4A). Numerous genes related to the DAM phenotype (*e.g.,* H2-D1, B2m, and others) were upregulated by HHcy, while homeostatic microglial markers (*e.g.,* P2ry12, Cst3, Fcrls, and others) tended to be downregulated (for a full list of HHcy sensitive genes in males, see Supplementary Table 4). KEGG functional pathways enriched for upregulated HHcy genes included virus infection (HIV, CMV, herpes virus, and hepatitis), proteolysis, oxidative phosphorylation, lysosomal, and NFκB signaling (Fig. 3B).

Differences between males and females were most apparent in the VIVIT treatment conditions (Fig. 4C and 4E). Unlike females, VIVIT-treated male mice in both diet groups (CT EGFP vs CT VIVIT, Fig. 4C); HHcy EGFP vs HHcy VIVIT, Fig. 4D) were associated with downregulation of numerous genes linked to DAM phenotypes (*e.g.,* H2-D1, H2-K1, B2m and others; for a full list of VIVIT-sensitive genes in male mice, see Supplementary Tables 5 and 6). Many of these were the same DAM markers upregulated by HHcy (Fig. 4A). In fact, most all of the genes (~ 90%) that were upregulated by HHcy (832 DEGs in the CT EGFP vs HHcy EGFP conditions) were also downregulated by VIVIT (1570 DEGs in HHcy EGFP vs HHcy VIVIT conditions) (Fig. 4G). KEGG functional pathways enriched for VIVIT-sensitive (downregulated) genes in both diet treatment conditions (Figs. 4D, 4F, and 4H) included multiple neurodegenerative diseases (*e.g.,* Alzheimer’s disease, prion disease, Parkinson disease, and Huntington disease), cardiomyopathy, phagosome/proteasome/lysosome, and reactive oxygen species linked to chemical carcinogenesis.

### Female vs male differences in microglial clustering patterns

**Figure 5:**
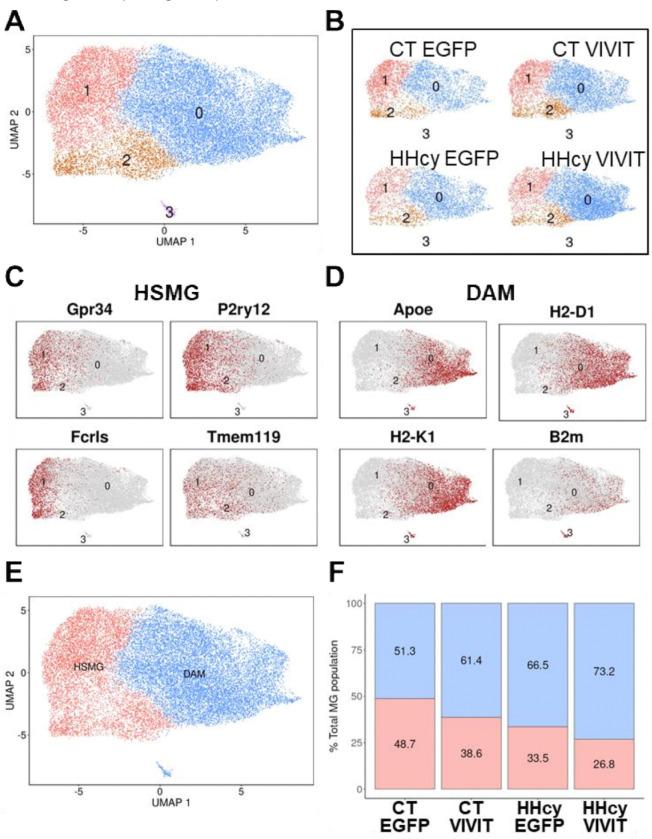
Microglial subclusters in female mice as a consequence of diet and AAV treatment. **A-B**, UMAPs showing six distinct microglia clusters across diet and AAV conditions for females. **C-D**, Expression of common HSMG (Gpr34, P2ry12, Fcrls, and Tmem119) and DAM markers (Apoe, H2-D1, H2-K1, and B2m) across the entire microglial cluster. **E**, Clusters were collapsed into two categories (HSMG and DAM) based on the distribution of cellular markers in **C**and **D. F**, Proportions of HSMG and DAM clusters (% total microglial (MG) population) across the diet and AAV treatment conditions. The DAM cluster was overrepresented in the HHcy conditions (especially HHcy VIVIT).

**Figure 6:**
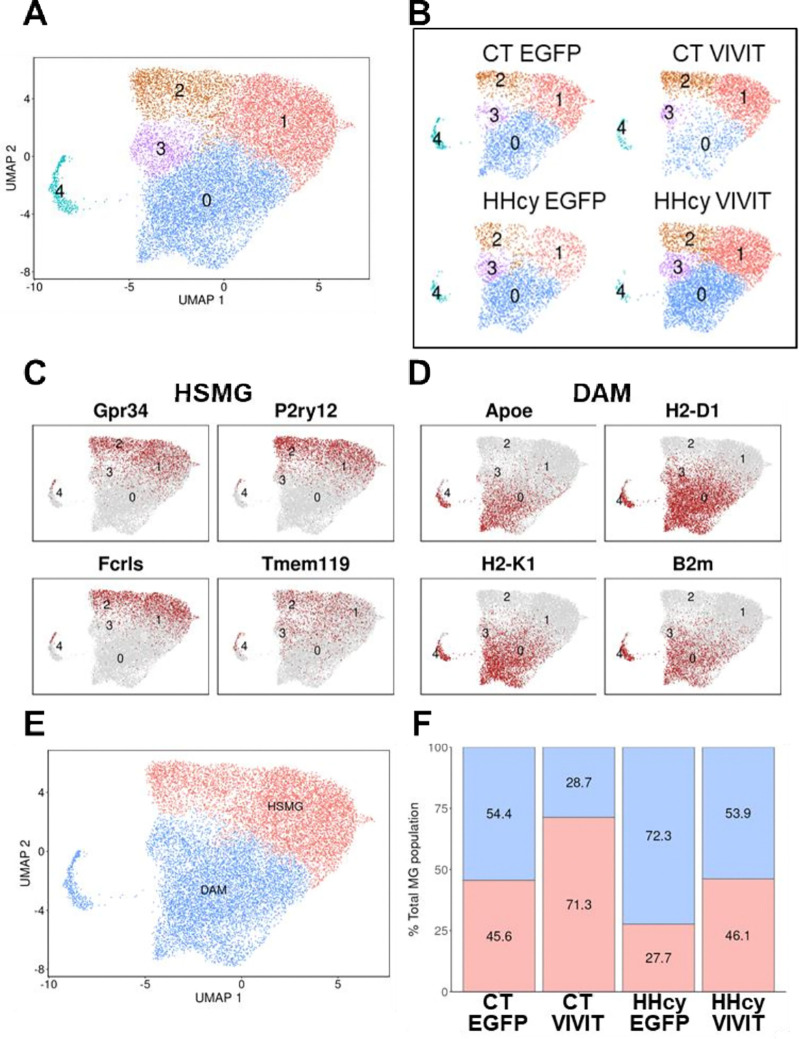
Microglial subclusters in male mice as a consequence of diet and AAV treatment. **A-B**, UMAPs showing five distinct microglia clusters across diet and AAV conditions for males. **C-D**, Expression of common HSMG (Gpr34, P2ry12, Fcrls, and Tmem119) and DAM markers (Apoe, H2-D1, H2-K1, and B2m) across the entire microglial cluster. **E**, Clusters were collapsed into two categories (HSMG and DAM) based on the distribution of cellular markers in **C** and **D. F**, Proportions of HSMG and DAM clusters (% total microglial (MG) population) across the diet and AAV treatment conditions. HHcy and VIVIT treatment had opposing effects on the distribution of DAM and HSMG clusters. DAM clusters were overrepresented, while HSMG clusters were underrepresented in the HHcy EGFP condition. In contrast, HSMG clusters were overrepresented and DAM clusters underrepresented in the CT VIVIT condition. There was a roughly equal distribution of DAM and HSMG clusters in the CT EGFP and HHcy VIVIT conditions.

We next looked at microglial clustering patterns in females (Fig. 5) and males (Fig. 6). There were four distinct microglial clusters in females (MG0-MG3) (Fig. 5A). But, unlike the males (see below), clusters in the female group seemed less sensitive to diet and AAV treatment conditions (Fig. 5B). The exception was cluster 0, which was more enriched in the HHcy VIVIT relative to the other conditions. The homeostatic microglial markers (Gpr34, P2ry12, Fcrls, Tmem119) tended to localize to clusters to 1 and 2 (Fig. 5C), while DAM markers (*e.g.,* Apoe, H2-D1, H2-K1, and B2m) localized to clusters 0 and 3 (Fig. 5D). Based on these observations, we collapsed microglial clusters into two broader categories (Fig. 5E): HSMG-like (clusters 1 and 2) and DAM-like (clusters 0 and 3), and the impact of diet and AAV on the proportions of these categories was evaluated (Fig. 5F). Clusters in the CT EGFP group were split roughly evenly across homeostatic and DAM categories (~ 52% DAM and, ~ 48% HSMG). The CT VIVIT, HHcy EGFP, and HHcy VIVIT conditions were associated with progressively greater proportions of DAM clusters (CT VIVIT 61.4%/38.6%; HHcy EGFP 66.5%/33.5%; HHcy VIVIT 73.2%/26.8%). The results suggest that both HHcy and VIVIT treatment tend to push microglial phenotypes to a DAM-like state in female mice.

Five distinct microglial clusters (MG0-MG4) were identified in males (Fig. 6A). But, unlike the females, clusters in the male group showed considerable variation across the different diet and AAV treatment conditions (Fig. 6B). Common HSMG markers including Gp34, P2ry12, Fcrls, and Tmem119 tended to localize with MG clusters 1 and 2 (Fig. 6C). In contrast, common DAM markers, like Apoe, H2-D1, H2-K1, and B2m tended to localize to MG clusters 0,3, and 4 (Fig. 6D). When clusters 1 and 2 were combined into a single category enriched in HSMG markers, and clusters 0, 3, and 4 were combined into a single category enriched in DAM markers (Fig. 6E), differences in clustering patterns were clearly discernible across diet and AAV treatments (Fig. 6F). The HHcy EGFP condition was underrepresented by HSMG clusters (27.7% total microglia) and overrepresented by DAM clusters (72.3% total microglia). The CT VIVIT condition showed nearly the opposite pattern: *i.e.,* CT VIVIT was enriched in HSMG clusters (71.3% of total microglia) and under-represented by DAM clusters (28.7% of total microglia). CT EGFP and HHcy EGFP showed roughly the same distributions (~ 45–46% homeostatic vs ~ 54% DAM). The results suggest that HHcy promotes the appearance of DAM microglia, while VIVIT helps maintain the presence of homeostatic microglia in males.

## DISCUSSION

The HHcy diet model exhibits numerous maladaptive VCID phenotypes including microhemorrhages and vascular inflammation, reduced cerebral blood flow and impaired neurovascular coupling, and cognitive loss in addition to synapse abnormalities[6–10]. Here, we show that HHcy also elicits possibly maladaptive disease-like transcriptional signatures in microglia. Notably, inhibition of reactive astrocyte signaling with AAV-Gfa2-VIVIT largely prevented or ameliorated these transcriptional changes in male, but not female, mice fed with HHcy-inducing diet. The results are consistent with work on multiple rodent models of disease that show improved outcomes after inhibiting reactive astrocyte signaling, but point to possible sex dependent differences in the way that astrocytes interact with microglia in the context of HHcy and/or small cerebral vessel disease.

### Microglial changes in response to HHcy

Neuroinflammation involving the production of numerous pro-inflammatory cytokines is a major feature of diet-induced HHcy[4]. The present study expanded these findings by showing the impact of HHcy on microglial subtypes defined by gene expression patterns. Consistent with previous work using RT-PCR to assess pro-inflammatory transcript levels [6, 8], HHcy-sensitive DEGs included antigen-presenting molecules (*e.g.,* H2-D1 and H2-K1), pro-inflammatory cytokine and chemokine species (*e.g.,* IL-15, CCL2, CCL4, and CCL5) (see Supplementary Table 4). Clustering patterns for microglia tended to shift away from more-homeostatic phenotypes (*e.g.,* characterized by high levels of P2ry12, Gpr24, and Fcrls) to the appearance of prominent clusters that expressed high levels of DAM markers including Apoe, H2-D1, and B2m (and others) (see Supplementary Table 4). Though initially defined in the 5xFAD mouse model of AD/amyloid pathology[32], DAM-associated genes or similar microglial subtypes have also been observed in human AD[33], multiple ADRDs, and/or ADRD mouse models[34–36]. Based on these reports, it’s not surprising that HHcy-sensitive DEGs in the present report (*e.g.,* see Fig. 3B) tended to be enriched in pathways for several human neurodegenerative diseases. Functionally, DAM phenotypes are thought to involve the mobilization of microglia to areas of pathology and/or the clearance of protein aggregates (like amyloid-β), cellular debris, or degenerating cells[37]. In the HHcy model, microglia may be responding to structural damage to astrocyte endfeet[7], vascular endothelial cells[38, 39], or other cell types at the blood brain barrier interface[12]. In addition, multiple C1q subunits (*i.e.,* C1qa and C1qb) are among the microglial genes induced by HHcy (see Supplementary Table 4), suggesting that microglia may be responding to degenerating synapses[40–43], which may contribute to the synaptic deficits observed in the HHcy diet model[10].

### Extensive crosstalk between microglia and astrocytes

Microglia and astrocytes exhibit extensive crosstalk and this bidirectional communication is a fundamental feature and/or cause of chronic glial reactivity found in most neurodegenerative conditions[44–47]. Activation of classic inflammatory regulators in astrocytes (*e.g.,* JAK/STAT, NFkB, and CN/NFATs) is robustly triggered by microglial derived factors, which, in turn, lead to the production and release of numerous factors that kindle and promote microglial reactivity[16, 48]. The CN/NFAT pathway is an attractive candidate mechanism for maintaining chronic neuroinflammatory signaling between glial cells because it produces numerous cytokines in response to Ca2 + dysregulation[49–51], which arises in multiple neural cell types at early stages of cognitive decline[52–55]. Levels and/or activity of CN/NFAT signaling constituents in humans are elevated in reactive astrocytes during Alzheimer’s disease[53, 56, 57], Parkinson’s disease[58], and cerebral small vessel disease[56, 59]. Inhibition of astrocytic CN/NFATs in intact animals has been shown to strongly modulate neuroinflammation and microglial reactivity in certain contexts[17, 60–62]. In the present report, we found that inhibition of astrocytic NFAT signaling limits transcriptional responses of microglia, particularly in male mice treated with HHcy diet, where VIVIT shifted microglial clusters from a more DAM-like state to a more homeostatic state. While the results of the present study, along with earlier reports, suggest that astrocytic CN/NFATs can directly affect astrocyte-microglia crosstalk, they cannot rule out possible indirect effects that may arise through other cell types. For instance, inhibition of astrocytic CN/NFATs has been shown to improve synapse function and/or normalize glutamate-mediated excitotoxicity in models of acute brain injury[18], chronic amyloid pathology[17, 19], and HHcy[10]. Healthier neurons may help keep microglia in a less reactive and/or more homeostatic state, whether astrocytes and microglia are talking to one another, or not. In addition, we previously showed that astrocytic CN/NFAT signaling was causatively linked to reduced cerebral blood flow and impaired neurovascular coupling[10], which may also affect the phenotypic state of microglia. And, of course, it’s highly feasible that the CN/NFAT pathway in astrocytes affects neurons and the cerebrovasculature by directly shifting microglia to a detrimental highly-reactive DAM-like state. The combination of interactions, including recruitment of other cell types (*e.g.,* oligodendrocytes and pericytes) is multitudinous. Clearly, further work is needed to determine the precise pathological sequelae associated with AD and ADRDs including the role of reactive astrocytes in initiating, maintaining, and/or resolving pathology.

### Sex differences in microglial phenotypes

Relatively little is known about interactions between HHcy and sex in relation to cardiovascular and or cerebrovascular pathologies. It’s clear that homocysteine levels increase with age and tend to be higher in men[63]. Moreover, HHcy is a strong risk factor for conditions like atherosclerosis[64, 65], coronary and peripheral artery disease[66, 67], cerebral hypoperfusion[9, 68], which occur more commonly in males versus females[69–72]. These observations suggest that HHcy may have a greater overall impact on brain health in males. However, the increased incidence of stroke, cerebral microbleeds, white matter hyperintensities and AD– all of which are linked to HHcy[5, 6, 73, 74] and appear more commonly in females[75–77]– would seem to run counter to this idea. These disparities highlight why additional research is needed to assess HHcy on native brain cells and neurologic function. Microglia are widely recognized for their central role in neuroinflammation, which is associated with most forms of acute brain injury, as well as chronic neurodegeneration. Mounting data demonstrates fundamental differences in microglia phenotypes (*e.g.,* numbers, morphology, and reactive state) between males and females during the lifespan[78], which could influence susceptibility to neurodegenerative diseases. Male mice have been shown to exhibit a heightened antigen-presenting phenotype[79] and may be more primed to respond to inflammatory events[80] compared to females. These observations are consistent with the sex differences we observed in response to HHcy diet. Specifically, far more microglial genes were upregulated in males versus females (926 vs 533, Fig. 4G and 3G, respectively) following HHcy exposure and males were more responsive to VIVIT, which has strong anti-inflammatory properties whether delivered to microglia[81] or astrocytes[82]. Microglial gene expression in females also did not follow protein expression as closely. For instance, P2ry12 gene expression was downregulated in females treated with HHcy diet (see Fig. 3A), while P2ry12 protein expression was elevated (see Fig. 1L).

Sex differences in microglial responses to both HHcy and VIVIT delivery could also arise from astrocyte-intrinsic factors, and/or sex differences in CN/NFAT signaling. Primary cortical astrocytes from male mice exhibit elevated production of inflammatory cytokines in response to an LPS insult[83]. And, in intact adult mice, astrocyte-specific inhibition of mGluR3 signaling had opposing effects on memory function in males and females (*i.e.,* females showed impairment, while males showed improvement). These observations suggest that astrocytes may have distinct roles in aging and/or disease-related cognitive decline, depending on sex. In regard to CN/NFAT signaling, estrogen/estradiol has been shown to protect against Ca2 + dysregulation in multiple contexts[84, 85], leading to reduced CN activity and/or suppression of CN-dependent processes[86–88]. In mouse heart, CN expression and CN-dependent NFAT activity are both higher in males versus females[89]. Interestingly, CN plays a causative role in cardiac hypertrophy which occurs more commonly in males[90]. Whether the blunted response of female microglia to both HHcy and VIVIT reflects a sex specific reduction in astrocytic CN signaling, due to the presence of estrogen (which could be a factor, considering the age of the mice investigated here) or other unknown sex-dependent factors, will require further investigation.

### Limitations of this study

The present study did not directly measure the molecular phenotypes of astrocytes. Though numerous reports have suggested that the CN/NFAT pathway directly modulates astrocyte reactivity[16, 50], our capacity to qualitatively and quantitatively assess the impact of either HHcy diet or AAV-Gfa2-VIVIT on reactive astrocyte subpopulations, and astrocyte gene expression signatures, was severely limited by the relatively few number of astrocytes that were collected. Another limitation of the study was that there was no vehicle control group or sham injection control group for assessing non-specific effects of AAV injection or injection injury on gene expression changes, which may have biased microglia phenotypes to a reactive or DAM-like state. Finally, because transcriptional analyses were limited to microglia, effects of reactive astrocyte signaling on other major cell types were not reported, but will be explored in follow-up studies.

## Conclusions

The present report is one of the first we know of to assess the impact of reactive astrocyte signaling on the transcriptional phenotypes of other major glial cells in intact animals. Amelioration of pro-inflammatory disease-like changes in gene expression and cell cluster patterns in microglia, via inhibition of the astrocytic NFAT pathway, suggests that reactive astrocytes play a major role in setting the inflammatory state of brain tissue in the context of cerebral small vessel pathology. Along with work from our lab and others, these findings continue to highlight the potential of astrocyte-targeting strategies to treat VCID, and perhaps AD, as well.

## Data Availability

The datasets used and/or analyzed during the current study are available from the corresponding author on reasonable request.

## Abbreviations

AAV: adeno-associated virus
AD: Alzheimer’s disease
ADRD: Alzheimer’s disease and related dementias
CN: Calcineurin
CT: Control
DAM: disease associated microglia
DEG: differentially expressed gene
HHcy: Hyperhomocysteinemia
HSMG: homeostatic microglia
KEGG: Kyoto Encyclopedia of Genes and Genomes
MG: microglial cluster
NFAT: Nuclear Factor of Activated T Cells
scRNAseq: single cell RNA sequencing
VCID: vascular contributions to cognitive impairment and dementia
